# Supplementary material for: Engineering Rubisco condensation in chloroplasts to manipulate plant photosynthesis
Source: Plant Biotechnol J. 2025 Mar 14;23(6):2140–9. doi: 10.1111/pbi.70047 (PMC12120884; doi:10.1111/pbi.70047)
Supplement: Supplementary file 1 — Figure S1 NtRubisco‐sfGFP purified from transgenic tobacco leaves showed obvious particle in vitro (Left); however, purified GFP (Middle) and NtRubisco (stained with Alexa Fluor® 647, Right) showed an even distribution in vitro. Scale bar: 10 μm. Figure S2 sfGFP‐triggered formation of Rubisco condensates remained unaltered when exposing to dark (top) or 1% CO2 (bottom). Scale bar: 10 μm. Figure S3 Transmission electron micrographs of leaf sections of WT and NtRubisco‐sfGFP plants. Figure S4 NtRubisco‐sfGFP showed a slightly larger cotyledon in early stage (15 days after sowing) on MS medium. (a, b) Plant images of NtRubisco‐sfGFP and WT, which were taken under blue light (a) and white light (b). (c, d) leaves identified from b by Image J (c) to calculate the cotyledon area (d). Figure S5 Quantification of Rubisco content in tobacco leaves by immunoblot analysis using an α‐RbcL antibody. The purified Rubisco from WT and NtRubisco‐sfGFP were quantified by Bradford method and loaded at different concentrations (a) to generate the standard curves (b). [file PBI-23-2140-s001.docx]

Supplementary Information


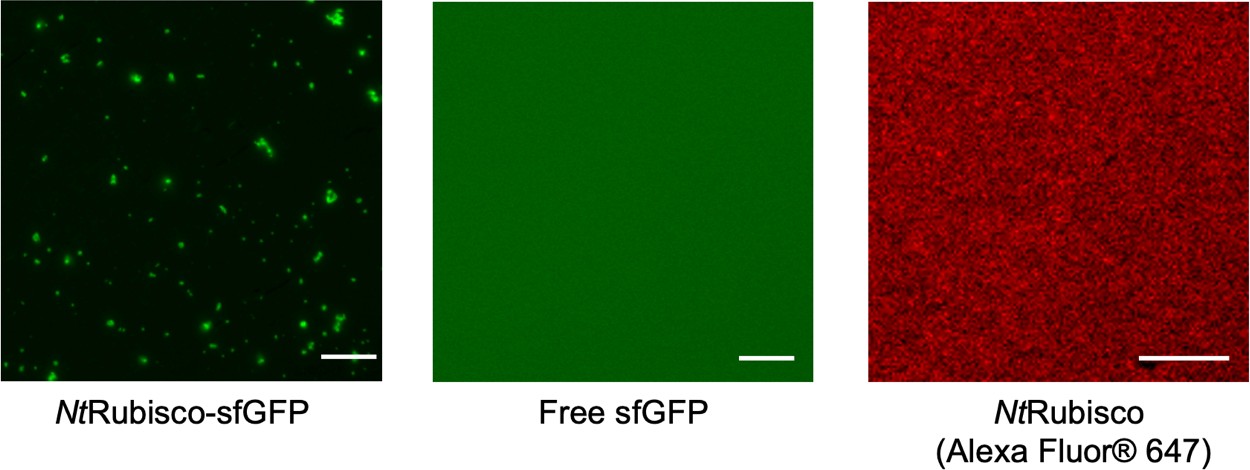


Figure S1. *Nt*Rubisco-sfGFP purified from transgenic tobacco leaves showed obvious particle *in vitro* (Left); however, purified GFP (Middle) and *Nt*Rubisco (stained with Alexa Fluor® 647, Right) showed an even distribution *in vitro*. Scale bar: 10 µm


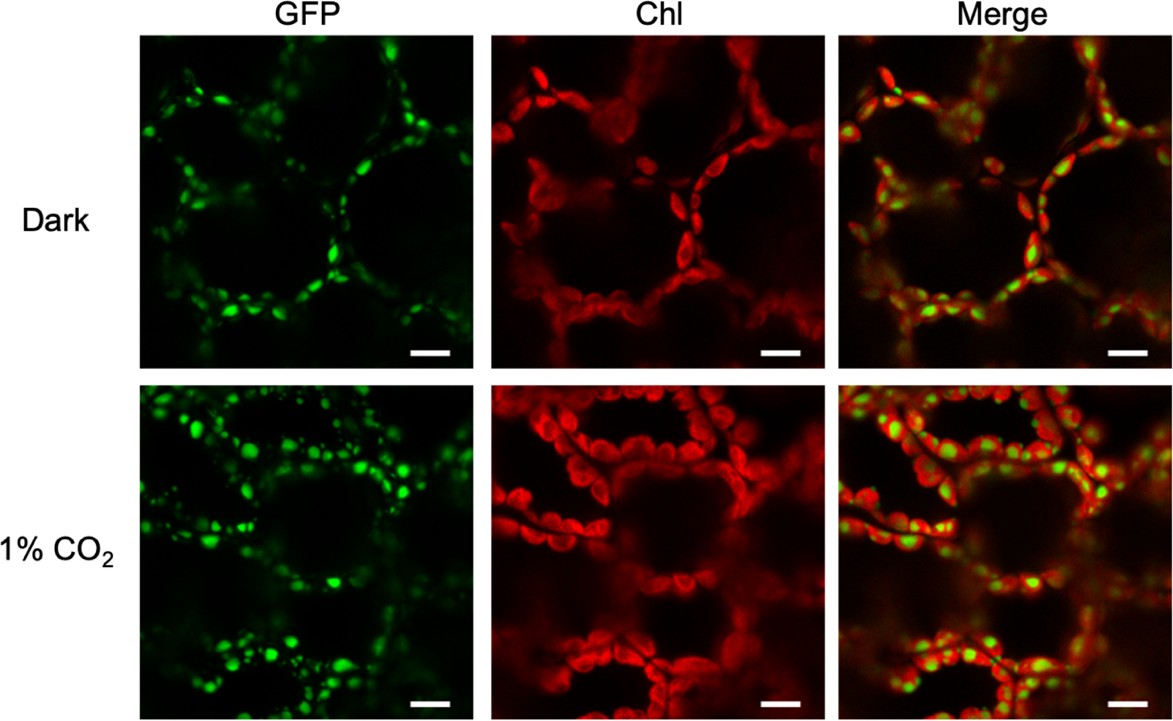


Figure S2. sfGFP-triggered formation of Rubisco condensates remained unaltered when exposing to dark (top) or 1% CO2 (bottom). Scale bar: 10 µm


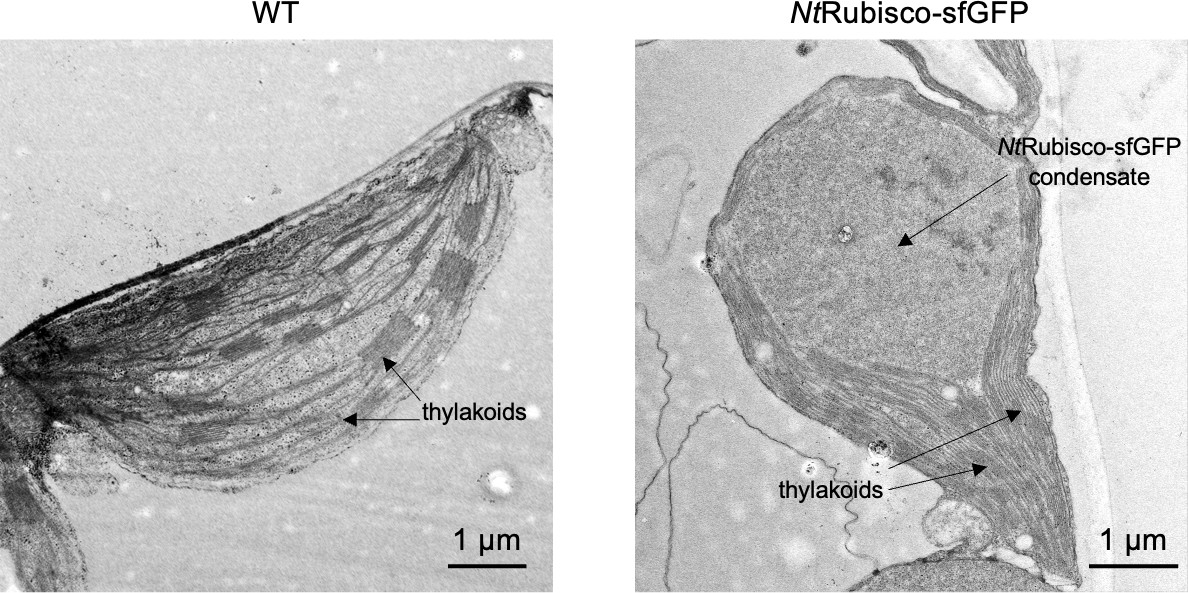


Figure S3. Transmission electron micrographs of leaf sections of WT and *Nt*Rubisco-sfGFP plants.


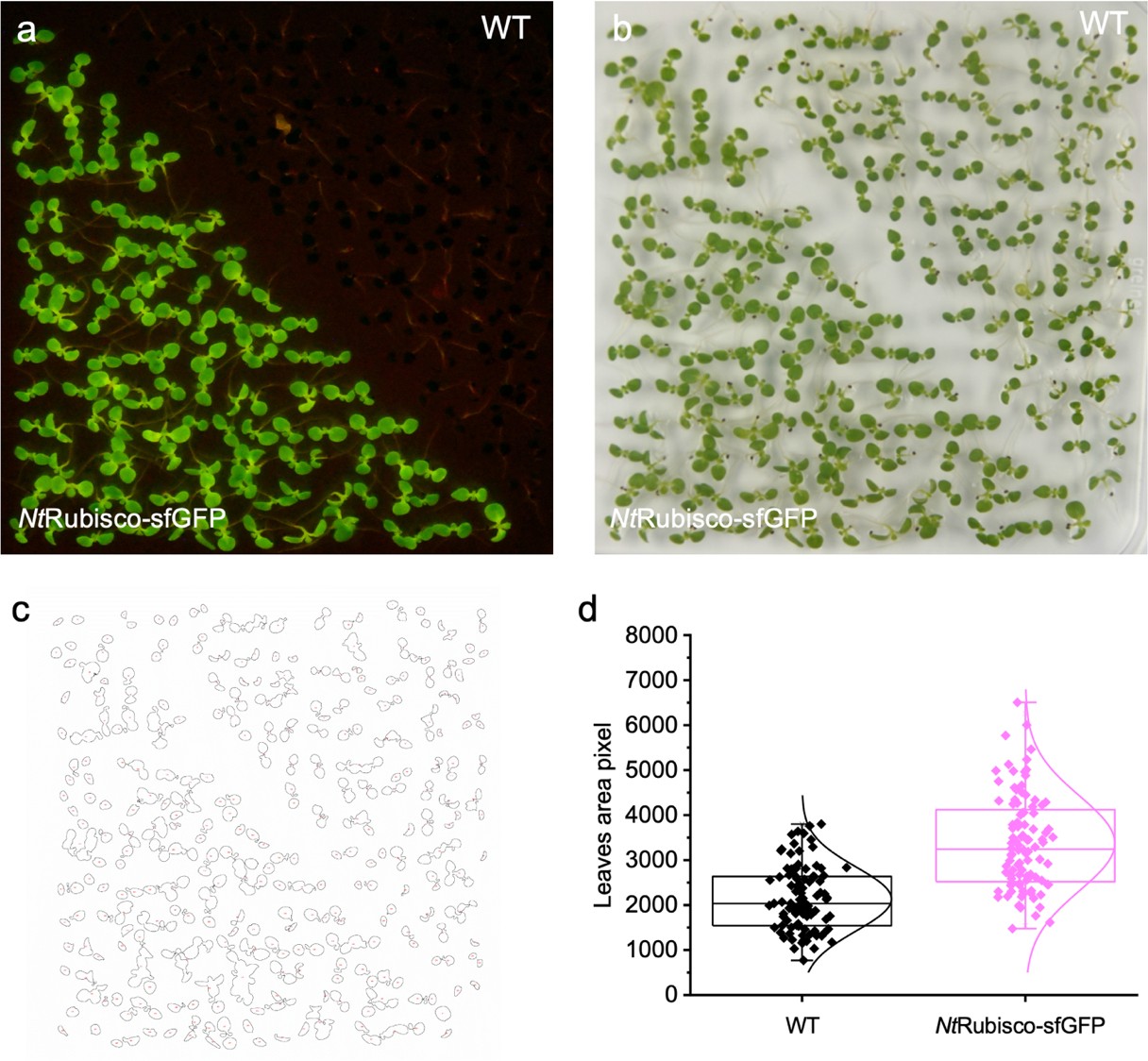


**Figure S4. *Nt*Rubisco-sfGFP showed a slightly larger cotyledon in early stage (15 days after sowing) on MS medium. a** and **b**, Plant images of *Nt*Rubisco-sfGFP and WT, which were taken under blue light (a) and white light (b). **c** and **d**, leaves identified from b by Image J (c) to calculate the cotyledon area (d).


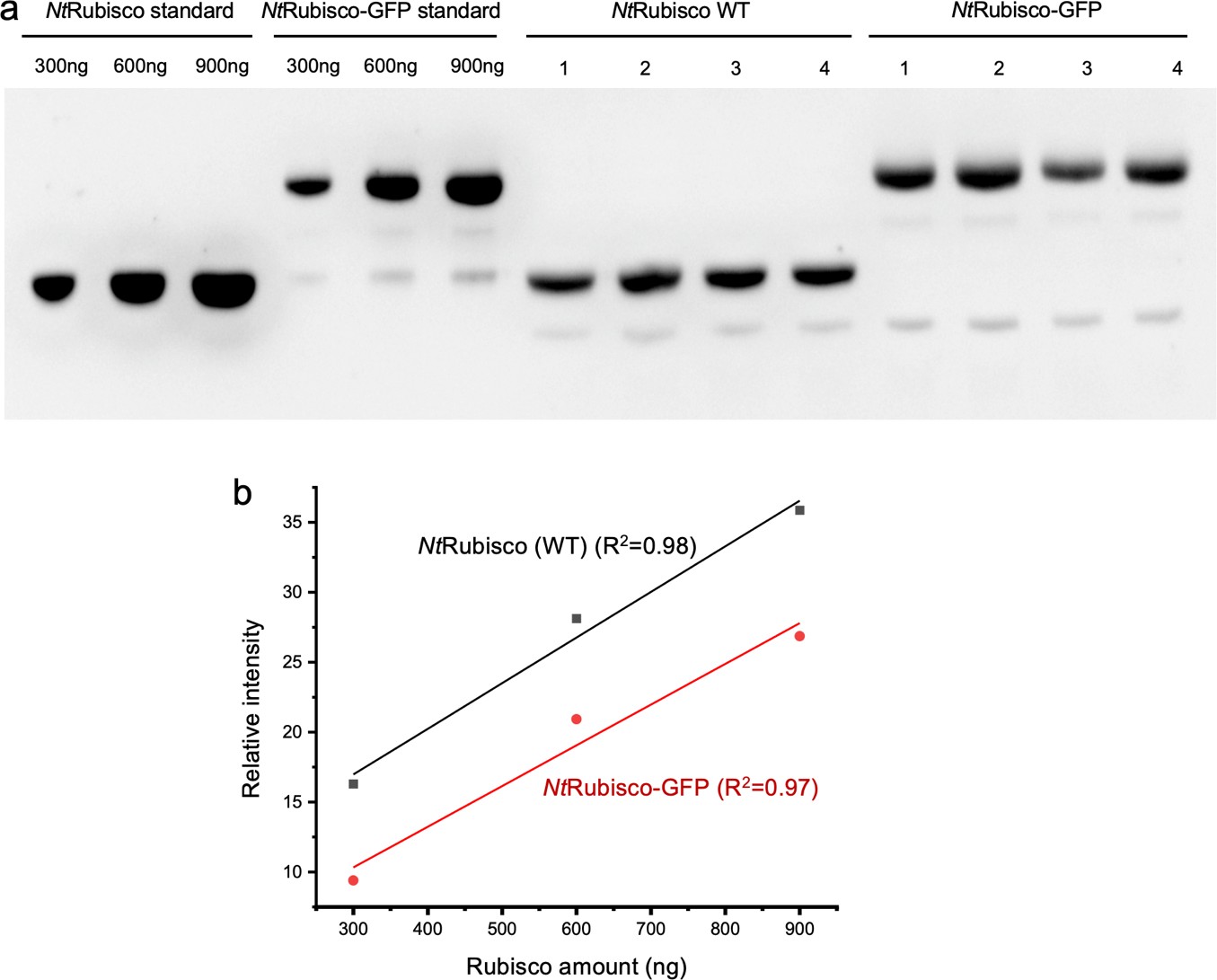


**Figure S5. Quantification of Rubisco content in tobacco leaves by immunoblot analysis using an α-RbcL antibody.** The purified Rubisco from WT and *Nt*Rubisco-sfGFP were quantified by Bradford method and loaded at different concentrations (a) to generate the standard curves (b).
